# Pronounced Diel Cycling of Dissolved Carbohydrates and Amino Acids in the Surface Ocean and across Diverse Regimes

**DOI:** 10.1021/acs.est.4c00491

**Published:** 2024-12-20

**Authors:** Theresa Barthelmeß, Antonia Cristi, Stacy Deppeler, Karl Safi, Karine Sellegri, Cliff S. Law, Anja Engel

**Affiliations:** † GEOMAR, Helmholtz Centre for Ocean Research Kiel, Kiel 24105, Germany; ‡ National Institute of Water and Atmospheric Research (NIWA), Wellington 6021, New Zealand; § National Institute of Water and Atmospheric Research (NIWA), Hamilton 3216, New Zealand; ∥ Université Clermont Auvergne, CNRS, Laboratoire de Météorologie Physique (LaMP), Clermont-Ferrand 63000, France; ⊥ Department of Marine Sciences, University of Otago, Dunedin 9016, New Zealand

**Keywords:** dissolved amino acids, dissolved carbohydrates, microbial turnover, diel organic matter cycling, phytoplankton production, viral lysis

## Abstract

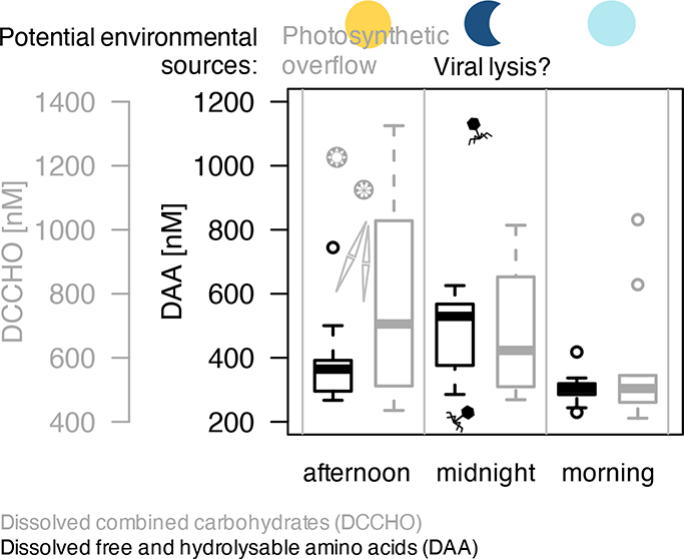

The metabolism of phytoplankton cells is synchronized with the diel light cycle. Likewise, associated heterotrophic bacteria adjust their diel expression of transporter- and catabolism-related genes to target the dissolved organic matter released by the phytoplankton cell. Dissolved combined carbohydrates (DCCHO) and dissolved amino acids (DAA) are major phytoplankton products and bacterial substrates. Here, we show that diel variations of DCCHO and DAA concentrations accounted for a significant turnover of the total organic carbon (TOC) pool (up to 5.0%, at a rate of 0.37 μM C h^–1^) and total organic nitrogen (TON) (up to 5.5%, 0.04 μM N h^–1^) across diverse oceanic regimes (sub-Antarctic to subtropical waters of the Southwestern Pacific Ocean). Glucose contributed most to the observed carbon turnover, while polar amino acids dominated the nitrogen turnover. DAA concentration and composition correlated with viral abundance, suggesting that viral lysis may have caused the the highest DAA concentration at night. Our finding of diel cycling of major dissolved organic phytoplankton products supports the notion of universally synchronized ecosystem dynamics. Such periodicity may enhance nutrient cycling and thus primary production and constrains parts of the yet uncharacterized labile organic carbon flux fueling the microbial carbon pump.

## Abstract 2

The diel cycling of major, dissolved organic phytoplankton products in the surface ocean is significant, rapid, and synchronized across diverse oceanic regimes, scheduling microbial turnover and potentially affecting ecosystem services such as the microbial carbon pump or marine biogenic aerosol formation.

## Introduction

Carbohydrates and amino acids are major phytoplankton products^1−3^ and contribute to the reactive, bioavailable components of marine dissolved organic matter (DOM).^4^ In contrast to energy storage molecules like carbohydrates (mainly polymeric glucose) or lipids,^5,6^ amino acids are essential for metabolic processes. Energy investment into their synthesis or acquisition is the most conserved process in phytoplankton cells, along with their heterotrophic bacterial associates.^7−9^ Phytoplankton metabolism is subjected to the diel light cycle and adjusts the allocation of photosynthetic energy over the course of a day. In the afternoon, phytoplankton production equals accumulation rates of energy storage molecules (in the form of carbohydrates) preparing for cell division.^10−12^ At night, transcriptomes for protein synthesis are prepared, causing an increase in the cell’s nitrogen-to-carbon ratio once the first sunlight is utilized for the biosynthesis of proteins and metabolic intermediates in the morning.^13^ It is further well-known that the diel synchronized metabolic cycle imprints particulate organic matter composition on the phytoplankton community level.^5,6,14^ Heterotrophic bacteria, which degrade dissolved organic phytoplankton products, align with the diel light cycle by upregulating the expression of transporter- and catabolism-related genes.^7,15,16^ Temporal niche-partitioning of a highly specialized bacterial consortium in the surface ocean suggests that carbon and nitrogen fluxes are decoupled.^17^ While new omics techniques shed light upon the intracellular, i.e., particulate level of diel metabolite production and degradation across the microbial trophic cascade, diel dynamics of the extracellular, dissolved pool of phytoplankton products are poorly understood.^15^

According to model simulations, two major phytoplankton sources supply dissolved, bioavailable products^18^ and may also respond to the diel light cycle: (1) healthy phytoplankton cells release DOM, including ecological signaling molecules and photosynthetic products. Signaling molecules can be neglected in terms of mass contribution.^15^ Glucose (Glc) as the major photosynthetic product constitutes 40% (up to 80%) of particulate organic carbon (POC) in surface ocean environments,^5^ and its production enhances toward dusk.^19^ It is hypothesized that excess photosynthetic products “overflow” once phytoplankton growth becomes limited by a lack of nutrients and/or cellular storage capacities are filled.^20^ In particular in the sun-lit surface, photosynthates may easily overflow, also as a mean to prevent photoinhibition.^13,21^ (2) Dead or dying phytoplankton cells release bioavailable DOM, which is triggered by lysis, senescence, or sloppy feeding.^18,22−24^ Marine viruses are susceptible to solar radiation and thus may shift their intracellular replication to daytime, while lytic events and subsequent viral infection occur predominately at night.^25−27^ Alternatively, excretion or egestion of DOM due to protist grazing^28^ alongside sloppy feeding by zooplankton^29^ may considerably alter the DOC flux.^30^ As zooplankton migration is navigated by the diel light cycle, it can induce the periodic release of bioavailable DOM.^23^

In the last decades, the main focus of the oceanic community has been directed toward the refractory dissolved organic carbon (DOC) reservoir due to its potential to store carbon over long time scales.^31,32^ It was commonly assumed that labile DOM in the surface ocean, which is cycled within minutes to days, cannot be observed to accumulate on the micromolar scale^31^ and is negligible in global carbon models as the gain and loss of labile DOM were assumed to be rather similar.^18^ Accordingly, most studies investigated oceanic depth profiles to assess the recalcitrant DOM flux in alignment with seasonal or global overturning circulations and, hence, vertical export.^33,34^ We found only a few studies investigating diel, dissolved carbohydrate and amino acid dynamics within the surface ocean environment.^35−37^ An increased diel resolution including nighttime observations may provide insights in particular into dissolved amino acid cycling.^35,36^ Although it was recently estimated that the supply of labile DOM results in a massive carbon flux of 15–25 Pg yr^–1^, corresponding to roughly a third of total annual primary production, the temporal resolution of rapid DOC cycling in the surface ocean remains coarse.^18^

Resolving diel dynamics of dissolved organic phytoplankton products in the surface ocean is also of interest for related research fields, such as atmospheric aerosol chemistry.^38^ Carbohydrates and amino acids are ubiquitous in ambient marine aerosols.^39−41^ Above productive regimes, the primary marine aerosol number and mass flux is enhanced and follows the diel light cycle.^42^ Once emitted from the ocean’s surface and lifted into relevant atmospheric heights, biogenic molecules including polymers of Glc and amino acids may enable cloud formation via ice crystallization or droplet condensation.^43−46^ The organic mass fraction in marine aerosols can explain a considerable part (35%) of the variability in cloud droplet numbers and, therewith, albedo above the Southern Ocean.^47^

This study investigates the diel cycling of the two major phytoplankton products (dissolved amino acids and combined carbohydrates) across four distinct regimes in the Southwestern Pacific Ocean. We examine whether the concentration of dissolved Glc increases with the accumulation of particulate Glc and thus follows the theory of photosynthetic overflow. We further hypothesize that the release of dissolved amino acids (DAA) is decoupled, as essential metabolites are lost during cell destruction. It was recently suggested that a major fraction of DOC resides at least once in the labile DOM pool.^18^ We thus expect to find evidence for a significant, yet uncharacterized, diel turnover of bioavailable DAA and dissolved combined carbohydrates (DCCHO) in the surface ocean. Based on the changes of DOM concentration over 24 h, we calculated rates and turnover times and characterized the molecular composition of cycled biopolymers.

## Material and Methods

East of Southern New Zealand, sub-Antarctic and subtropical waters converge along the Chatham Rise, creating filaments and eddies, which provoke intense phytoplankton blooms year-round.^48^ Diverse trophic conditions are encountered within short distances.^49^ The Sea2Cloud voyage onboard RV Tangaroa (TAN2003) was conducted from March 15 to 27, 2020, in early austral autumn. Surface water samples from the Tangaroa underway flow-through system were collected every 8 h at midnight (mi, *n* = 11) local time (NZDT), 08:00 AM (am, *n* = 10), and 04:00 PM (pm, *n* = 10). Sunrise and sunset in March are at approximately 07:30 AM and 07:50 PM. The time points for sampling were chosen to represent different metabolic phases of the phytoplankton cell cycle as suggested by Halsey and Jones (2015):^13^ (i) phytoplankton production equals the accumulation of storage carbohydrates (pm); (ii) after cell division at dusk, temporary energy resources are catabolized during the night (mi); (iii) solar radiation is directed into the biosynthesis of proteins in the morning (am). Regimes were categorized according to salinity and nutrient concentrations, which were measured along the cruise track via the underway system (inlet at ∼6 m depth),^50^ separating the region into four distinct regimes i.e., sub-Antarctic waters (SAW, *n* = 10), subtropical waters (STW, *n* = 3), subtropical frontal waters (STF, *n* = 12), and a mixed regime (Mix, *n* = 6) (*N* = 31; Figure S1). Sample sizes were smaller for total organic nitrogen (TON) and community composition (Table S1). Six samples were collected from a working boat instead of the underway system. These samples did not differ from the regular underway samples at a given time point of the day (*Wilcoxon* test, package stats 3.6.3, command applied “wilcox.test”, comparison of DAA, DCCHO, and TOC concentration was executed for morning and afternoon samples) and were those integrated to complete the data set.

In brief, Chl *a* concentration was fractionized by filtration and analyzed by spectrofluorometry. Samples for phytoplankton, bacterial, and viral abundances were fixed with glutaraldehyde (GDA), stained with SYBR Green II for groups without autofluorescence, and enumerated by flow cytometry (Supporting Information). Samples for the determination of amino acids (4 mL) and combined carbohydrates (17 mL) were stored at −20 °C until analysis by high-performance liquid chromatography (HPLC, 1260 HPLC system Agilent) and high-performance anion-exchange chromatography (HPAEC) in combination with pulsed amperometric detection (PAD) (Dionex ICS-3000), respectively.^51−53^ Samples for the dissolved phase were filtered through 0.45 μm Acrodisc filters (Pall Corporation) before storage, including DAA and DCCHO, while samples of the total phase were filled directly into the glass vials, i.e., total amino acids (TAA) and total combined carbohydrates (TCCHO). DAA samples thus include the complete pool of free plus hydrolyzable amino acids. Only the combined fraction of dissolved carbohydrates was assessed as the free fraction (<1 kDa) is lost during dialysis, which is necessary to get rid of the salt ions. 13 amino acids and 12 carbohydrates were separated on a *Kinetex* Core–Shell C18 LC column and a *Dionex* CarboPac PA10 IC column, respectively. However, muramic and gluconic acids were below the detection limit. Due to the hydrolysis, the amino acids glutamic acid and aspartic acid cannot be distinguished from glutamine and asparagine and are therefore summarized as GlX and AsX, respectively. Analytical precision was calculated as the relative standard deviation (SD) between analytical replicates. Replicates of DCCHO and TCCHO samples deviated by a relative SD of 2.9 ± 2.5% and 2.1 ± 1.5%, respectively. Replicates of DAA and TAA samples deviated by a relative SD of 2.7 ± 2.0% and 6.9 ± 5.5%, respectively. The detection limit for combined carbohydrates and amino acids was 5–10 nM and ∼1 nM, respectively.^51,52^ The recovery rate was above 90% for combined carbohydrates.^51^ Particulate amino acids and combined carbohydrates (PAA/PCCHO) were calculated by subtracting the dissolved from the total phase. Combined samples for total nitrogen (TN) and total organic carbon (TOC) (20 mL) were acidified with 20 μL of 32% HCl (Certified AR for Analysis, Fisher Chemical) and stored at 4 °C. Samples were analyzed by high-temperature catalytic oxidation (TOC-VCSH, Shimadzu) after Sugimura and Suzuki (1988).^54^ Analytical replicates of TOC samples deviated by 1.2 ± 0.5%. The detection limit of TOC was 1 μM. Total organic nitrogen (TON) was estimated by subtracting inorganic nitrate (NO_3_) and ammonium (NH_4_) concentrations from TN.^50^ All sampling glass vials were combusted at 500 °C for 8 h before usage.

Statistics were calculated in RStudio (Version 1.4.1106). Basic analysis of data included the calculation of the mean (M) and SD, and quoted numbers apply to this format (M ± SD) if not stated otherwise. Variables were tested for whether they were normally distributed (*Shapiro–Wilk* test, package stats 3.6.3, command applied “shapiro.test”) and homogeneous in their variance (*Levene’s* test, package car 3.1–1, command applied “leveneTest”). Most variables exhibited a non-normal distribution, but only a few variables significantly diverged from homogeneous variances, most importantly PCCHO. To evaluate whether the two factors, i.e., regime and time of the day, had a significant effect on the distribution of data, we applied a nonparametric and multifactorial analysis of variances (ANOVA).^55^ To account for the non-normal distribution, the data were aligned and rank transformed (art, nonparametric analysis), in accordance with the factors and before evaluating their effects (multifactorial design) (package ARTool 0.11.1, command applied “art”). Finally, a type III ANOVA was applied to account for the unbalanced design (package car 3.1–1, command applied “anova”). Whether concentrations were sgnificantly influenced by the factor of interest (time of the day) was tested by performing a post hoc test based on the *Tukey* method (package ARTool 0.11.1, command applied “art.con”). For each regime, the diel periodicity of organic matter cycling was estimated with the help of a generalized additive model (GAM) and an integrated smoothness estimator (package mgcv 1.8-31, command applied “gam” and “predict”).^56^ GAM fits are based on the original data. To represent the diel periodicity in DOM cycling, a cyclic cubic regression spline with four knots was selected (Figure 2a–d). Based on the 8 h sampling intervals, we can only represent simplified and symmetric DOM curves.

Median turnover rates d(*n*)/d(*t*) of DCCHO and DAA were calculated for those regimes, in which at least three data points per time spot were available, i.e., for the STF and SAW. The median concentration characterizing the daily minimum [*n*]_min_ was subtracted from the median concentration marking the daily maximum [*n*]_max_ (eq 1). The change in concentration d(*n*) is represented in Figure 2e–h. The change in time d(*t*) is 16 and 8 h for DCCHO and DAA, respectively. For calculating combined turnover rates of DCCHO plus DAA in carbon and nitrogen equivalents, the change in time was set to 12 h (Table S4).
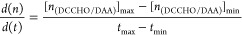
1

Turnover (*X*) is defined as the ratio of the diel change in DAA and DCCHO concentration d(*n*) over the mean TOC or TON concentration *n*_(TOC/TON)_ (eq 2).
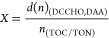
2

We performed principal component analysis (PCA) to evaluate the state of degradation (which is dependent on the molecular composition of DAA) and whether it corresponded to any of the two factors. The data set was centered and normalized before the PCA was computed (package stats 3.6.3, command applied “prcomp”). Major variance within the data set is reflected on the first axis (principal component), accounting for 42.8% of the total variance. It is commonly interpreted that the first axis represents differences in degradation, along which several amino acids decode for fresh organic matter (e.g., leucine, phenylalanine), while others mark degradation (e.g., alanine, glycine, gamma-aminobutyric acid).^34,57,58^ To compute the degradation index, it is necessary to compare as many data points as possible to obtain the most reliable results. Therefore, available DAA data from the TAN2003 voyage were integrated, including not only the 31 samples presented here but also other samples that were collected from the sea surface microlayer (*n* = 7), surface waters (*n* = 1), and the air–sea interaction tanks (ASITs) (*n* = 21).^50^ These samples are represented in gray and marked as “other” (Figure 3a). The degradation index was calculated after the approach of Kaiser and Benner (2009).^34^ A second tool was applied to estimate the fractions of DAA in percent, which can be assigned to the labile (degraded within hours to days), semilabile (within months to decades), and refractory (within centuries) organic matter pool.^57^ An absolute concentration of 85.5 nM DAA represents the refractory pool, while the semilabile concentration is assumed to vary in relation to DOC (here TOC), however, at a constant ratio (max. suggested DAA-carbon yield for the refractory plus semilabile pool: 1.6%).^57^ The labile fraction is then represented by the remainder of encountered DAA surface concentrations and yields (Supporting Information). To assess the correlation between DAA concentration, the degradation index, and virus particle abundance, nonparametric *Spearman* rank correlation tests were performed (package stats 3.6.3, command applied “cor.test”). *Spearman* correlations are indicated by the coefficient *rho*.

## Results

### Regimes

This study was conducted in the Southwestern Pacific Ocean in the proximity of New Zealand (Figure S1). Within the study area, subtropical waters (STW) and sub-Antarctic waters (SAW) are characterized by low primary production due to low concentrations of macronutrients (STW) or micronutrients (SAW). The convergence of these two water masses eliminates nutrient deficiencies within the subtropical front (STF). Mixing of the Cook Strait currents with STW creates a further distinct regime (Mix), which is replenished in nutrients. The water masses were delineated according to variations in surface salinity and nutrients.^50^ Total chlorophyll *a* (Chl *a*) concentration was significantly different between regimes, with the highest in the STF (1.94 ± 0.67 mg m^–3^), followed by the Mix (0.84 ± 0.21 mg m^–3^), and declined in the STW (0.40 ± 0.09 mg m^–3^) and SAW (0.39 ± 0.08 mg m^–3^) (art ANOVA, *p*-value <0.001). Chl *a* concentration of microphytoplankton (>20 μm) and nanophytoplankton (2–20 μm) was significantly elevated in the STF in comparison to the other regimes (Figure S2 and Table S2) (art ANOVA, *p*-value <0.001). Chl *a* concentration of picophytoplankton (0.2–2 μm) was highest in the STF and Mix and lowest in the STW and SAW (art ANOVA, *p*-value <0.001). Nanophytoplankton cells were most abundant in the STF and SAW (art ANOVA, *p*-value <0.001). Eukaryotic picophytoplankton showed maximal abundance in the Mix regime, while lowest abundances characterized the STW. *Synechococcus* spp. abundance was maximal in the STF, followed by the Mix regime, and declined to a minimum in the SAW. Bacterial abundance was significantly different across regimes as highest concentrations were reached in the STF (3.30 ± 0.84 × 10^6^ cells mL^–1^) and Mix, and lowest in the SAW and STW (1.71 ± 0.41 × 10^6^ cells mL^–1^) (art ANOVA, *p*-value = 0.003). Cell abundance did not change significantly in the course of the day for any of the represented groups (Figure S2 and Table S2). Within these diverse conditions, the diel cycling of DCCHO and DAA as major phytoplankton products was investigated.

### Combined Carbohydrates

**Figure 1:**
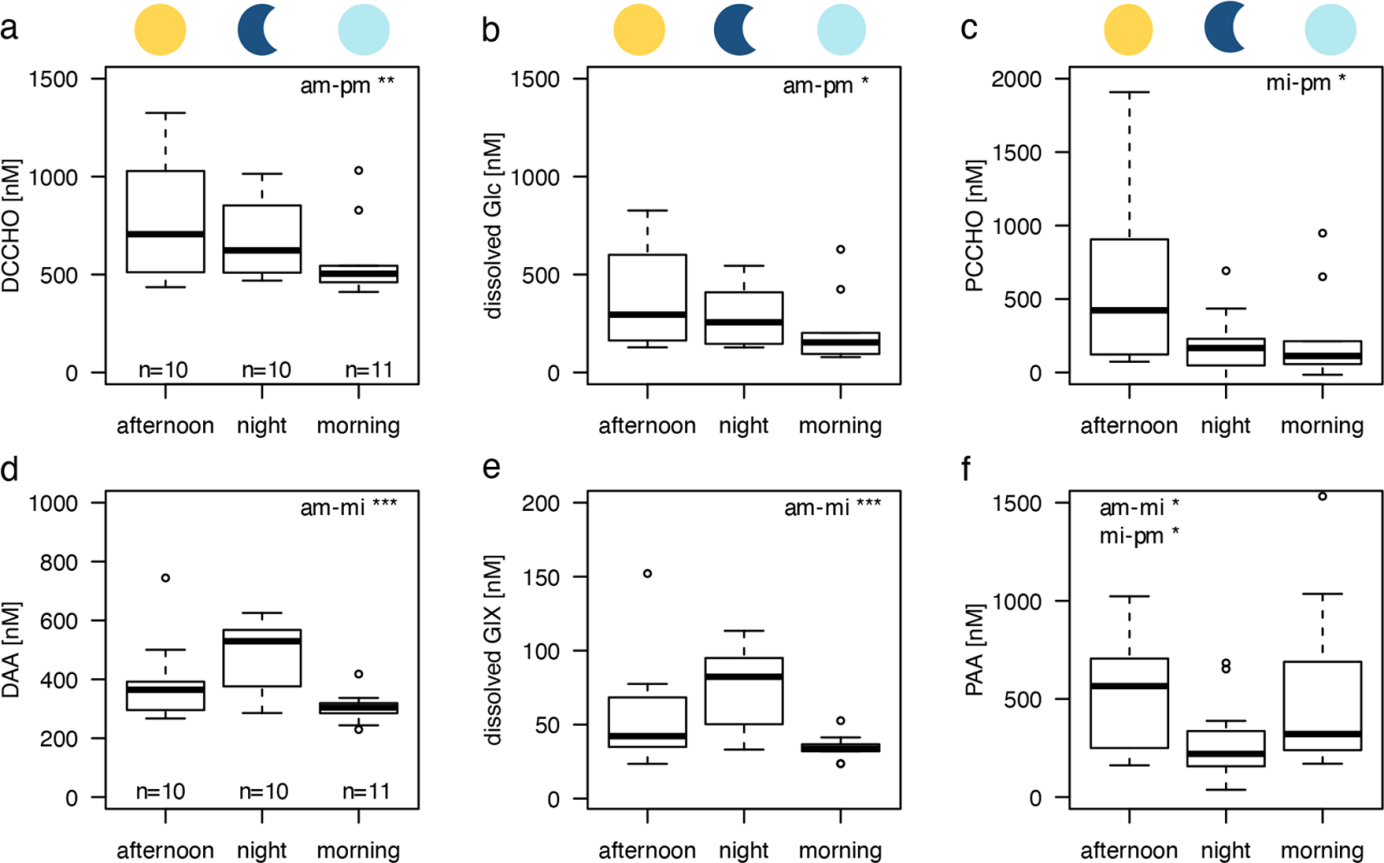
Diel organic matter cycling of (a) dissolved combined carbohydrates (DCCHO), (b) glucose (Glc) as the major molecular contributor to DCCHO, (c) particulate carbohydrates (PCCHO), (d) dissolved amino acids (DAA), (e) glutamic acid (GlX) as the major molecular contributor to DAA, and (f) particulate amino acids (PAA) averaged across regimes. Note that the applied scales differ. Colored symbols represent the time of the day, the afternoon is yellow (04:00 PM), midnight (12:00 PM) dark blue, and the morning (08:00 AM) is light blue. Pairwise post hoc comparisons (*Tukey* test) revealed significant differences between concentrations in the course of the day. Asterisks decode the level of significance. Sample numbers of each time point are indicated below the first column of plots (a,d) but apply for all variables presented in the following plots (b,c,e,f).

**Figure 2:**
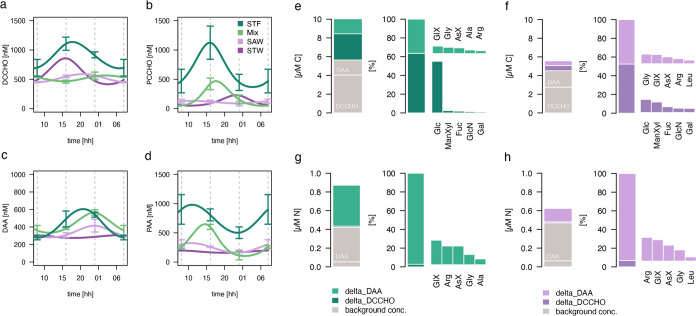
(a–d) Estimated diel organic matter cycling in the four regimes is depicted in dark green (subtropical front, STF), light green (mixed waters, Mix), purple (subtropical waters, STW), and lilac (sub-Antarctic waters, SAW). (a) Dissolved combined carbohydrates (DCCHO), (b) particulate combined carbohydrates (PCCHO), (c) dissolved amino acids (DAA), and (d) particulate amino acids (PAA) cycling is depicted. Note that the applied scales differ. The time of the day, as represented by the *x*-axis, starts at 08:00 AM and progresses until the next morning. Dotted lines indicate real sampling events at the respective times. Standard error bars for each regime (except STW) and sampling time were derived from the real data set used to estimate the development of concentrations over the course of the day. The number of samples per regime and time point are as listed in bracelets (STF: pm, mi, and am = 4; Mix: pm, mi, and am= 2; SAW: pm, mi = 3, am = 4; STW: pm, mi, and am = 1). (e–h) Median concentration of DCCHO and DAA in carbon and nitrogen equivalents turned over within a day (delta). Background concentrations represent the remainder of DCCHO and DAA concentrations, which did not vary over the course of the day. Delta was calculated for (e,f) carbon, and (g,h) nitrogen within the subtropical front (STF, green) and the sub-Antarctic regime (SAW, lilac). The contributions of DCCHO and DAA are divided into subgroups, of which the five major components are indicated in percent. Average concentrations of these major components per regime and time point are listed in Table S3. (g,h) DCCHO contribution to nitrogen turnover was negligible and is not depicted. Amino acids: glutamic acid (GlX), aspartic acid (AsX), arginine (Arg), alanine (Ala), glycine (Gly), leucine (Leu), and serine (Ser). Carbohydrates: glucose (Glc), fucose (Fuc), mannose/xylose (ManXyl), glucosamine (GlcN), and galactose (Gal).

On average, DCCHO exhibited a maximum in the afternoon (786 ± 326 nM) and reached a background concentration of 570 ± 200 nM the next morning (Figure 1a) (art ANOVA, *p*-value = 0.010). Afternoon concentrations differed significantly from morning DCCHO concentrations (*Tukey* test: am–pm: *p*-value = 0.007), as also reflected in its main molecular contributor Glc (Figure 1b; *Tukey* test: am–pm: *p*-value = 0.012). Particulate combined carbohydrates (PCCHO), calculated as the difference between the dissolved and total phases, also exhibited a diel periodicity. In alignment with DCCHO, the highest PCCHO concentration was observed in the afternoon (579 ± 587 nM), while it subsided to its daily minimum at night (236 ± 312 nM) (art ANOVA, *p*-value = 0.034; Figure 1b). Afternoon concentrations differed significantly from nighttime PCCHO concentrations (*Tukey* test: mi–pm: *p*-value = 0.042). The magnitude of cycling varied between regimes, as depicted in Figure 2 by means of the GAM fit. DCCHO cycling within the STF and STW was greatly pronounced in comparison with the other regimes (Figure 2a). Within the Mix regime, DCCHO concentration increased only slightly toward night. The diverging cycles also resulted in significant concentration differences across regimes. The highest mean DCCHO concentration was observed in the STF (903 ± 261 nM), while lowest (517 ± 64 nM) occurred in the Mix regime (art ANOVA, *p*-value <0.001, Table S2). The large standard deviations of the averaged concentrations can be explained by the different magnitudes of cycling across regimes. The STF exhibited the highest PCCHO production, while PCCHO cycling was dampened in the Mix regime (Figure 2b). The diel cycling of DCCHO was accompanied by changes in its molecular composition (Table S3 and Figure S3a–d). As the major molecular component of DCCHO, Glc also defined diel cycling as it accounted for an average of 43.7 Mol% in the afternoon and declined overnight to only 32.8 Mol% in the morning. All other fractions reflected the relative loss in Glc, i.e., increased slightly from the afternoon until the next morning. The only exception was glucuronic acid (GlcX), which reached its highest relative share at night.

### Amino Acids

Unlike carbohydrates, DAA reached their maximum concentration on average at night (477 ± 126 nM). In alignment with DCCHO again, DAA concentration declined to its minimum concentration in the morning (305 ± 52 nM) and only slightly increased during the day (art ANOVA, *p*-value <0.001; *Tukey* test: am–mi: *p*-value <0.001; Figure 1d). The main molecular component contributing to a diel cycling in DAA concentration was GlX (*Tukey* test: am–mi: *p*-value <0.001; Figure 1e). A reciprocal pattern became apparent for particulate amino acid (PAA) concentration (Figure 1f), as it declined significantly at night (276 ± 218 nM), but reached concentrations as high as in the afternoon (537 ± 299 nM) again the next morning (art ANOVA, *p*-value = 0.009). Afternoon and morning concentrations differed significantly from nighttime PAA concentration (*Tukey* test: am–mi: *p*-value = 0.031; mi–pm: *p*-value = 0.013). Moreover, DAA concentration differed significantly across regimes (art ANOVA, *p*-value = 0.008). Averaged DAA concentrations (STF: 437 ± 157 nM; Mix: 436 ± 109 nM) and diel cycling were maximal and of similar magnitude in the STF and the Mix regime, whereas averaged DAA concentration was lowest in the STW (290 ± 26 nM) and a diel cycle was absent (Figure 2c). PAA concentration and the cycling magnitude were also significantly different between regimes, with highest concentrations in the STF (737 ± 352 nM) and lowest in the STW (176 ± 25 nM) (art ANOVA, *p*-value <0.001) (Figure 2f).

**Figure 3:**
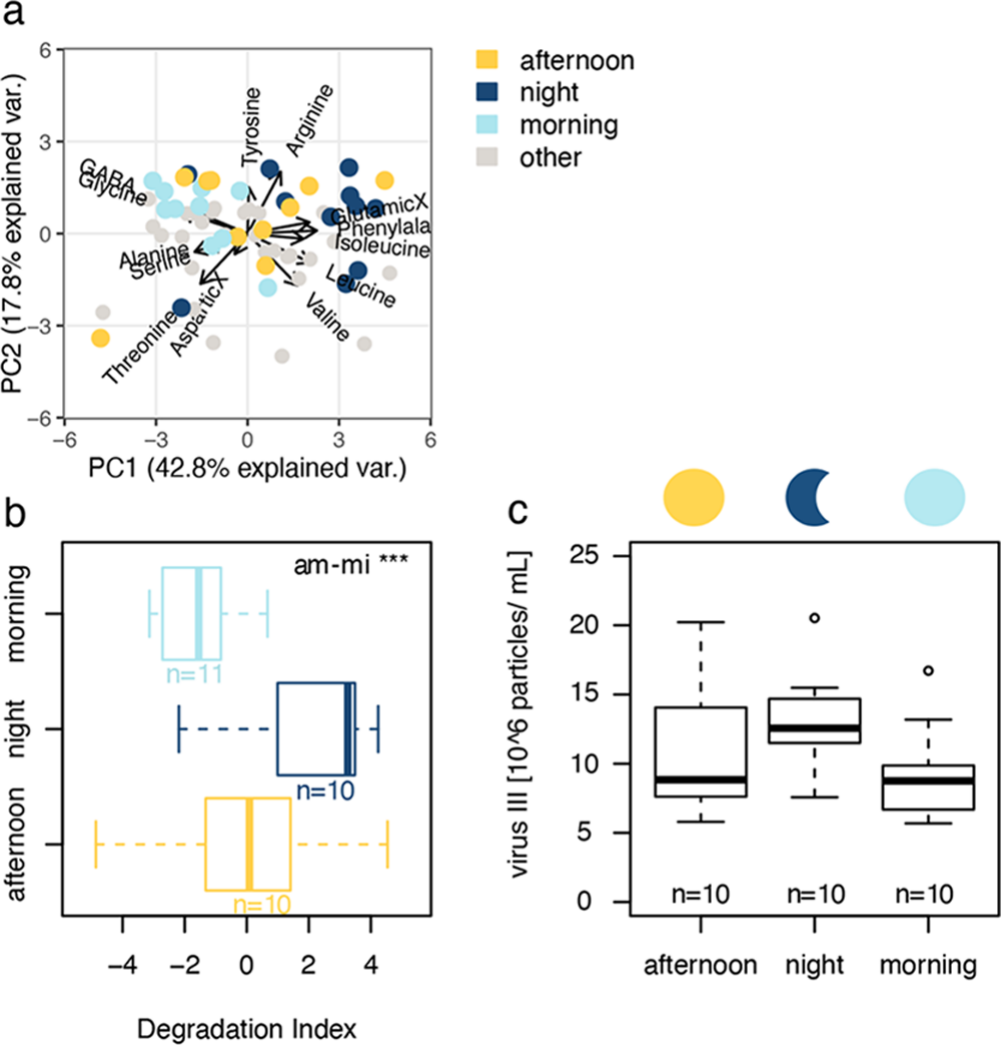
(a) Differences in the molecular composition of dissolved amino acids (DAA) are shown by means of a principal component analysis (PCA). Major variance occurs along the first axis and represented compositional changes dependent on the time of the day. (b) Degradation indices (DIs) derived from the PCA. (c) Virus particle counts averaged across regimes. Midnight samples (12:00 PM) are depicted in dark blue, the afternoon in yellow (04:00 PM), and the morning in light blue (08:00 AM). Pairwise post hoc comparisons (*Tukey* test) revealed significant differences between DIs in the course of the day. Asterisks decode for the level of significance.

DAA composition was dominated by glycine (Gly; 25 Mol%) and aspartic acid (AsX; 17 Mol%), followed by GlX (13 Mol%), while smaller fractions were comprised of arginine (Arg; 4.2 Mol%) or leucine (Leu; 3.5 Mol%) (Table S3). Changes in the relative diel DAA composition were evident (Figure S3e–h) and are also captured by the first PCA axis (representing 42.8% of variance) (Figure 3a). At night, DAA was comprised, in particular, of a higher fraction of dissolved GlX but was also characterized by phenylalanine (Phe), isoleucine (Iso), Leu, and valine (Val). In the morning, the molecular composition shifted toward a higher relative share of Gly, alanine (Ala), serine (Ser), and gamma-aminobutyric acid (GABA). The corresponding degradation indices are represented in Figure 3b. Explicitly for pivotal amino acids defining degradation profiles (e.g., Ala, Gly, Phe, and Leu), our PCA loadings of the first axis corresponded in magnitude and direction to loadings derived from a marine data set, which was assembled to reflect the range of degraded and fresh DOM.^34^ Degradation indices changed significantly over the day (art ANOVA, *p*-value = 0.001; *Tukey* test: am–mi: *p*-value <0.001), exhibiting positive values at night (2.0 ± 2.3) and negative values in the morning (−1.6 ± 1.2). Significant differences were also established between regimes (art ANOVA, *p*-value = 0.038). A further quantitative tool was applied, which assigns organic matter lability based on the DAA-carbon yield in relation to DOC concentrations (DAA-C yield).^57,59^ It is reasonable to assume that TOC presents DOC fairly well and can be introduced as a proxy for DOC (Supporting Information). While DAA-C yields were on average 2.56 ± 0.82% at night, they declined to 1.54 ± 0.33% in the morning (art ANOVA, *p*-value = 0.009; *Tukey* test: am–mi: *p*-value <0.008), matching the suggested range of a DAA-C yield representative of semilabile substrate (1.1–1.6%^59^). Accordingly, the labile fraction of the DAA concentration declined to approximately 0% in the morning and increased to 31% at night. Regimes did not significantly influence the DAA-C yields.

Moreover, we enumerated viral particle counts by flow cytometry and were able to discriminate three size classes, of which category III contained the smallest particles (<0.22 μm). Virus particle (III) abundance changed with the time of the day (Figure 3c), exhibiting the highest abundance at night (13.1 ± 3.4 × 10^6^ particles mL^–1^) and the lowest abundance in the morning (9.3 ± 3.6 × 10^6^ particles mL^–1^). The highest viral abundance (III) was measured in the Mix regime (12.8 ± 5.2 × 10^6^ cells mL^–1^) and the STF (11.6 ± 4.0 × 10^6^ particles mL^–1^), while the lowest abundance characterized the STW (6.4 ± 1.1 × 10^6^ particles mL^–1^). However, temporal and regional differences in virus particle abundance were not significant (Table S2). *Spearman* rank correlations revealed further that the daily increase in DAA concentration at night and its subsequent decline aligned well with virus particle abundance (III) (rho = 0.49, *p*-value = 0.006) (Figure S4a). In contrast, virus particles (III) did not correlate with DCCHO concentration. Moreover, virus particle abundance (III) was significantly correlated with degradation indices (rho = 0.46, *p*-value = 0.011; Figure S4b), which reflect the molecular composition of DAA.

### Dissolved Organic Matter Turnover

We focused on the dissolved phase and its composition to decipher organic matter turnover. Median rates, at which DOM was turned over, were calculated for the SAW and STF, representing low and high productivity regimes (Table S4). The Mix and STW regimes were omitted from analysis due to limited sample size. DCCHO exhibited a median turnover rate of 5.8 nM Glc h^–1^ within SAW (34.9 nM C h^–1^). This was low in comparison to the STF, where the median turnover rate was 29.1 nM Glc h^–1^ (174.3 nM C h^–1^). DAA were cycled at rates of 63.2 nM C h^–1^ in the unproductive SAW, while increasing to 199.7 nM C h^–1^ in the productive STF. Combining DCCHO and DAA translates into carbon turnover rates of 88.6 and 365.6 nM C h^–1^ in the SAW and STF, respectively. Dissolved Glc alone contributed 14% and 57% to carbon turnover, while DAA accounted for 48% and 34% in the SAW and STF, respectively (Figure 2e,f). A smaller fraction of the diel carbon turnover can be attributed to the residual carbohydrates, i.e., 38% and 9%, respectively. Cycled dissolved carbon concentration, including DAA and DCCHO, accounted for a considerable fraction of TOC: In the SAW and STF, 1.6% and 5.0% of TOC was turned over on a daily basis, respectively. Nitrogen was cycled at rates of 12.9 nM N h^–1^ (SAW) and 37.5 nM N h^–1^ (STF) with the most prominent fractions being represented by the amino acids GlX (SAW: 23%; STF: 26%), Arg (SAW: 25%; STF: 20%), and AsX (SAW: 17%; STF: 20%), while DCCHO nitrogen turnover was negligible (Figure 2g,h). Interestingly, DAA turnover was greatest for amino acids that exhibit the largest topological polar surface area (GlX, Arg, and AsX). Nitrogen turnover summed up to 2.7% and 5.5% of TON per day for the SAW and STF, respectively.

## Discussion

### Photosynthetic Overflow Triggers the Release of Dissolved Glucose in the Afternoon

We assessed whether the accumulation of particulate photosynthetic products in the surface ocean, prominently represented by Glc, is concomitant with an increase in its dissolved concentration. We expected to see that both phases increase toward the afternoon as phytoplankton cells store chemical energy equivalents in preparation for metabolizing them at night when they become deprived of solar energy.^11,14^ In general, our data set appears to support the “overflow” hypothesis;^20^ DCCHO and PCCHO concentrations were maximal in the afternoon, declined to their minimum until the next morning, and Glc dominated DCCHO composition (Figure 1a–c). However, DCCHO and PCCHO dynamics were fully synchronized only within the STF (Figure 2a,b, dark green). In the least productive STW, dissolved Glc concentration exhibited a peak in the afternoon, but PCCHO concentration remained low (purple). In contrast, the Mix regime was characterized by an afternoon increase in PCCHO concentration only (light green). DCCHO and PCCHO oscillation was completely absent in the SAW (lilac). We propose that these differences across regimes resulted from the additive effects of photosynthetic balancing, encountered nutrient conditions, community composition, and trophic interactions. Depending on the cell surface-to-volume ratio and its membrane permeability, a certain amount of uncharged organic molecules, such as Glc, may passively leak out,^60^ whereas active exudation of larger polymers is controlled by the phytoplankton cell.^61,62^ Not only cell size but also taxonomy influences the direction and magnitude of photosynthetic overflow. While eukaryotic phytoplankton cells are able to allocate photosynthetic products into lipids, i.e., redirecting Glc production, cyanobacteria rely on (polymeric) Glc as energy storage molecules.^6,63^ In the STW, photosynthetic overflow was thus likely enhanced as a consequence of severe nutrient deficiency and the smaller cell sizes of the encountered phytoplankton community (Table S2), which favored increased leakage in the absence of an alternative pathway to store or invest excess energy. Phytoplankton cells are further known to exude polysaccharides, which can aggregate into particulate matrices.^64^ Polysaccharide matrices can serve as protective agents, e.g., fighting viral infection by entrapping infected phytoplankton cells and virions,^65,66^ and/or stimulate trophic interactions, as these aggregates attract bacteria and grazers.^65,67,68^ “Selfish” bacteria mask the dissolution of substrate as they are capable of allocating the enzymatic breakdown of large polysaccharides into their periplasm.^69^ A masked or simply delayed dissolution of extracellular PCCHO could thus explain the diel dynamics observed in the Mix.

### Night-Time Peak in Labile Amino Acids Potentially Caused by Viral Lysis

We further hypothesized that the release of DAA is decoupled from DCCHO dynamics. Indeed, DAA concentrations were maximal at night and thus decoupled from DCCHO release (Figure 1d–f). PAA and DAA dynamics were inverse, suggesting that cell destruction caused an increase in DAA concentration. This was corroborated by viral particle abundance, which increased at night and exhibited the same diel periodicity as DAA concentration (Figure 3). Virions infecting small, unicellular organisms are capable of rapid replication,^70^ and viral particles found in oceanic waters likely reflect active virus–host interactions.^26^ In particular, cyanophages are known to exhibit a clear diel cycle of intracellular replication (afternoon) and subsequent lysis and infection (night).^25,26^ GlX contributed the largest share to carbon and nitrogen turnover within the pool of amino acids (Figure 2e–h). Besides its osmotic properties in the phytoplankton cytosol,^100^ GlX also serves as a universal biomolecule channeling the acquisition of inorganic nitrogen.^71,72^ As intracellular and extracellular nitrogen stocks are exploited in order to assemble viral progenies, viral lysates contain high amounts of amino acids.^71,73^

The generalized additive model fit reproduced the highest nighttime DAA release in the productive waters of the STF and Mix regime (Figure 2c,d, green), aligning well with the highest observed viral particle abundance, picophytoplankton abundance, Chl *a* concentration (0.2–2 μm), *Synechococcus* spp., and bacterial abundances (Table S2). Contrastingly, the lowest viral particle, picophytoplankton, and bacterial abundances were measured in the STW (purple), where the DAA concentration did not oscillate. Viral infection and subsequent lytic events are predicted to be proportional to the population density of host cells, implying that high lytic DOM fluxes occur especially in productive regimes.^74^ Viral infection thus likely caused abundant picophytoplankton cells to lyse at night in the STF and Mix, and to a smaller extent in the SAW. In the Southern Ocean, viral lysis has been reported to trigger more than half of the daily carbon loss, which was equivalent to half of the daily gross carbon production. However, the remainder was lost due to grazing activity.^22^ Therefore, we cannot exclude that other factors, such as grazing, initiated phytoplankton cell destruction, which ultimately led to the observed DAA release.

### A Substantial Fraction of TOC Is Cycled during One Day

We expected to find evidence for a significant yet uncharacterized diel turnover of bioavailable carbon and nitrogen in the surface ocean. Indeed, the observed diel oscillation in DCCHO and DAA concentration proposed periodic phytoplankton production and release in concert with rapid bacterial cycling. However, rapid bacterial cycling would require that released phytoplankton products are bioavailable, i.e., labile. Glc was the main compound controlling the diel shift in the DCCHO concentration. Among neutral sugars, Glc concentration declined the fastest in bacterial degradation experiments amended with fresh phytoplankton DOM.^75^ We have further introduced proxies to assess the state of microbial degradation from labile to refractory based on DAA concentration and composition.^34,59^ In summary, contrasting degradation indices and a decrease in labile substrate followed the nighttime peak in DAA concentration (Figure 3b, Supporting Information). The observed diel cycling of DCCHO and DAA can thus likely be attributed to bacterial degradation. Heterotrophic bacteria are capable of rapidly consuming larger polymers within minutes due to membrane embedded transporters for oligosaccharides thus waiving prior extracellular hydrolysis.^76^ In addition, energy storage molecules cleaved into Glc monomers by extracellular enzyme activity are hydrolyzed at maximal rates of 22–34 nM Glc h^–1^.^5,69^ The turnover rate calculated for the STF (29.1 nM Glc h^–1^) is thus within the reported range (Table S4). Only a few heterotrophic bacterial uptake rates of dissolved hydrolyzable amino acids (DHAA) have been reported,^77,78^ of which maximal rates were 28.3 nM N h^–1^. DHAA uptake rates accounted for up to 62% of the bacterial carbon demand during productive periods, however, were assessed in a freshwater system, which may differ from its marine equivalent.^78^ Uptake rates of dissolved free amino acids (DFAA) typically range between 3.8 and 35.3 nM N h^–1^ in marine environments.^77,79^ Considering both substrates (DFAA and DHAA), uptake rates match well in magnitude with the maximal turnover rate calculated for the STF (37.5 nM N h^–1^) (Table S4).

Diel TOC turnover (including cycled DCCHO and DAA concentrations; Figure 2e,f) was 5% within the STF. Phytoplankton cells usually represent a minor fraction of TOC and are part of the POC pool. On average, 2% of TOC is substituted by POC.^4^ This fraction may rise to approximately 15% in productive oceanic waters.^80^ The here observed diel TOC turnover (5% of TOC) is thus significant once put into perspective, as it may hold one-third of the standing phytoplankton stock (up to 15% of TOC). Our results are in line with model simulations, which were based on average annual primary production rates of the global surface ocean (63 Pg C yr^–1^;^18^).^81^ Moran et al. (2022) concluded that roughly one-third of phytoplankton products (15–25 Pg C yr^–1^) flow directly into the labile DOC pool. The diel turnover of dissolved organic phytoplankton products, as assessed in our study, thus likely contributes to the yet unresolved labile DOC flux.^18^

### Implications for Global Biogeochemical Cycling and Climate Feedbacks

Our results indicate that the diel TOC turnover was dominated by dissolved Glc and GlX. The transformation from simple, bioavailable phytoplankton products, such as Glc and GlX, into persistent, refractory molecules has been suggested to be rapid and to relate directly to primary production.^82,83^ Microbially processed DOC is characterized by the absence of heteroatoms (nutrients), its low molecular weight, and its chemical complexity,^4,82,84^ but a minor fraction of the refractory DOC is still constituted by Glc or amino acids.^33,34^ The transformation process describes the microbial carbon pump, which is estimated to sequester approximately 0.18 Pg C yr^–1^.^85^ We do not have any direct evidence of this transformation process, however, we observed that across diverse regimes DCCHO and DAA declined to minimal and least variable concentrations in the morning (Figure 1a,d). Furthermore, the respective DAA-C yield (1.54%) reflected the threshold set for the refractory plus semilabile organic matter pool (1.1–1.6% DAA-C yield).^59^ Semilabile organic matter is turned over within months to years,^59^ while the refractory pool is basically inert to microbial processing.^86^ We further propose that DOM release was caused by photosynthetic overflow and viral lysis. The “viral shunt” contributes to the functioning of the microbial carbon pump as viral lysis channels biomass away from higher trophic levels and redirects it into microbial degradation.^32,87^ In a multitrophic model, it was assessed that in phytoplankton communities with viruses, primary production and phytoplankton diversity are significantly stimulated due to enhanced dissolved organic nitrogen fluxes and regenerated growth.^74^ Whether viral lysis or protist and zooplankton grazing^23^ caused DAA concentration to peak at night, we cannot say. In more general terms, the destruction of phytoplankton cells still stimulates DOM release, heterotrophic degradation, and regenerated growth, thus fueling the microbial carbon pump.^32,85^ Any perturbations of this large labile organic carbon flux originating from photosynthetic balancing or the destruction of cells may alter the size of the ocean’s refractory reservoir within decades.^18^

The interdisciplinary goal of our research campaign Sea2Cloud was to investigate the role of biogeochemistry in marine aerosol formation.^50^ Interestingly, we discovered increased secondary aerosol formation at night in particular in the STF and potentially involving the marine precursor species nitric oxide (NO).^88^ The production of NO can be traced back to, e.g., rapid DAA degradation by heterotrophic bacteria,^89,90^ and the reaction pathways of phytoplankton cells to environmental stressors such as viral lysis or grazing.^91,92^ Improved understanding of source dynamics linked to the oceanic emission of biogenic aerosols and the formation of clouds is required.^38^ Resolving rapid organic matter cycling in the surface ocean is thus a step towards deciphering air–sea interaction processes and potential climate feedbacks.

In summary, we found synchronized diel oscillations in DCCHO and DAA concentrations across various regimes. It has been suggested that the surface ocean ecosystem follows universal diel cycles, which influence trophic interactions, and biogeochemical cycling.^7,16,25^ This has climate relevant implications, as pronounced diel cycling of organic matter may impact e.g. the functioning of the microbial carbon pump and cloud formation over the Southern Hemisphere oceans. Future, more detailed measurements of their quality, quantity, and drivers should be carried out to resolve whether such synchronized diel fluxes are ubiquitous across the surface ocean.

## Supporting Information Available

Additional details on phytoplankton methods; turnover and degradation indices; sample size and data (PDF)

The data sets presented in this study can be found in an open-access online repository: https://doi.pangaea.de/10.1594/PANGAEA.974276.^94^

## Abbreviations

DOM: dissolved organic matter
TOC: total organic carbon
DOC: dissolved organic carbon
POC: particulate organic carbon
Chl *a*: chlorophyll *a*
DCCHO: dissolved combined carbohydrates
DAA: dissolved amino acids
PCCHO: particulate combined carbohydrates
PAA: particulate amino acids
DAA: dissolved amino acids
PCCHO: particulate combined carbohydrates
PAA: particulate amino acids
GlX: glutamine/glutamic acid
AsX: asparagine/aspartic acid
Arg: arginine
Leu: leucine
Iso: isoleucine
Phe: phenylalanine
Val: valine
Ala: alanine
Ser: serine
GABA: gamma-aminobutyric acid
Gly: glycine
Glc: glucose
Fuc: fucose
ManXyl: mannose/xylose
GlcN: glucosamine
Gal: galactose
SAW: sub-Antarctic waters
STW: subtropical waters
STF: subtropical frontal waters
Mix: mixed waters
