# Supplementary material for: Pronounced Diel Cycling of Dissolved Carbohydrates and Amino Acids in the Surface Ocean and across Diverse Regimes
Source: Environ Sci Technol. 2024 Dec 20;59(1):419–29. doi: 10.1021/acs.est.4c00491 (PMC11741105; doi:10.1021/acs.est.4c00491)
Supplement: Supplementary file 1 — es4c00491_si_001.pdf [file es4c00491_si_001.pdf]

SUPPLEMENTARY INFROMATION

Pronounced diel cycling of dissolved carbohydrates and amino acids in the surface ocean and across diverse regimes

Authors: Theresa Barthelmeß[1]*, Antonia Cristi[2], Stacy Deppeler[2], Karl Safi [3], Karine Sellegri[4], Cliff S. Law[2,5], and Anja Engel[1]

[1]GEOMAR, Helmholtz Centre for Ocean Research Kiel, 24105 Kiel, Germany.
[2]National Institute of Water and Atmospheric Research (NIWA), 6021 Wellington, New Zealand.
[3]National Institute of Water and Atmospheric Research (NIWA), 3216 Hamilton, New Zealand.
[4]Université Clermont Auvergne, CNRS, Laboratoire de Météorologie Physique (LaMP), 63000 Clermont-Ferrand, France.
[5]Department of Marine Sciences, University of Otago, 9016 Dunedin, New Zealand.

*Corresponding author
Email: tbarthelmess@geomar.de

List of content

Figure S1. Map of stations sampled, p. S1.
Figure S2. Regime dependent community composition, p. S2.
Figure S3. Relative molecular change in DCCHO and DAA over the course of the day and across regimes, p. S7.
Figure S4. Data on the dependence of DAA concentration and degradation index on viral particle abundance, p. S4.

Table S1. Number of samples, p. S5.
Table S2. Overview of averaged concentrations, p. S5.
Table S3. Overview of averaged concentration of the main molecular contributors to diel turnover, p. S6.
Table S4. Overview of values and literature used to calculate diel turnover, p. S8.

Methods, p. S1 -S6.
Discussion, p. S6.
References, p. S9.

SUPPLEMENTARY INFORMATION

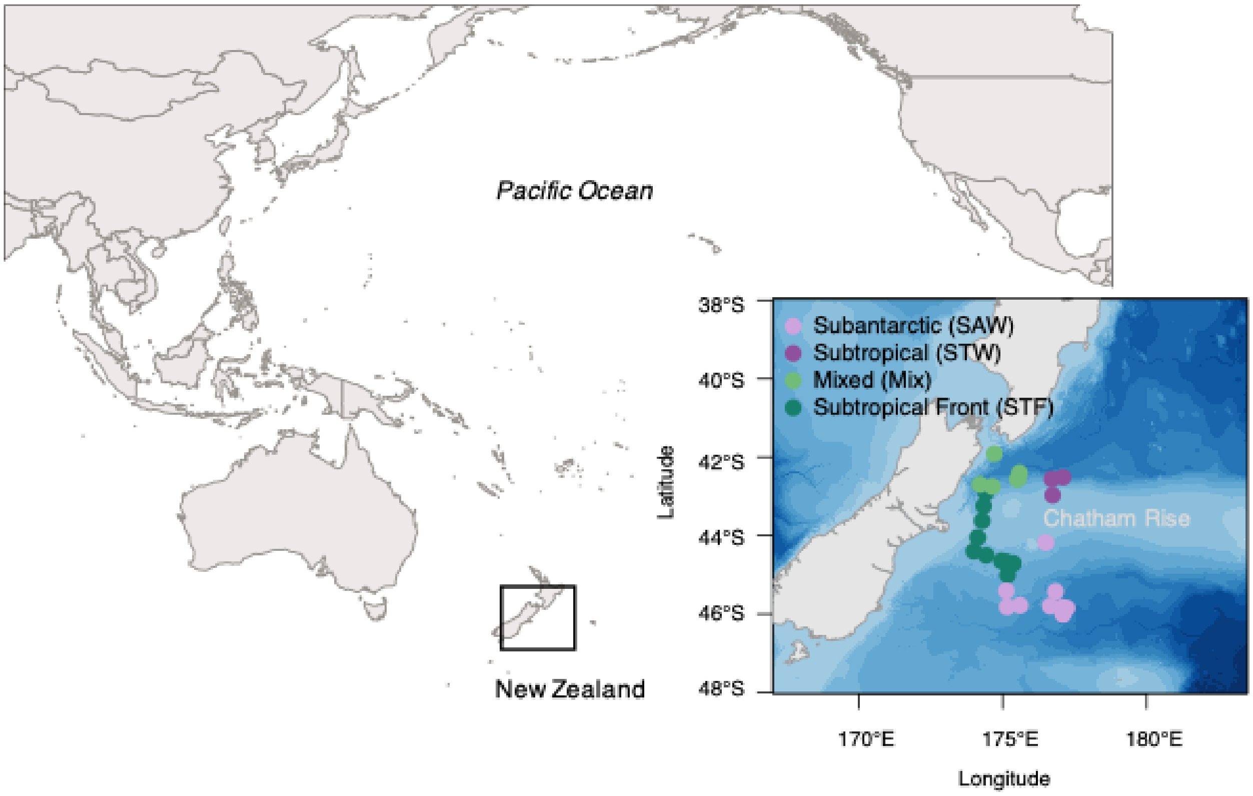

Figure S1. Stations along the cruise track (TAN2003) divided into the four regimes as encountered in the South Pacific Ocean. East of New Zealand, subtropical and sub-Antarctic water masses converge along the Chatham Rise. Green represents nutrient replete while purple represents nutrient deplete conditions. Blue contours decode for depth.

METHODS

Microbial community

Fractionized Chl *a* concentration, phytoplankton and bacterial abundances were analyzed. Chl *a* concentration was fractionized into particles larger than 20 µm, 2-20 µm and 0.2-2 µm by filtering 250 mL seawater sequentially through 47 mm polycarbonate filters. Filters were stored at -80°C until analysis. Chl *a* was extracted with 90% acetone and measured by spectrofluorometry (Varian Carey spectrofluorometer). Total Chl *a* concentration represents the sum of its fractions. Samples for phytoplankton and bacterial abundance were fixed with 0.5% glutaraldehyde (GDA), flash frozen and stored at -80°C, until analyzed by flow cytometry (Accuri™ C6 Plus, BD Biosciences). Phytoplankton size groupings corresponded to the size fractioning of Chl *a* concentration: Nano-phytoplankton included cells larger than 2µm, while pico-phytoplankton cells were smaller than 2µm. Bacterial cells were stained with SYBR Green II. Eukaryotic phytoplankton and prokaryotic picoplankton (*Synechococcus* spp.) were identified through their auto-fluorescent pigments. Viral particle counts were analyzed with the same method as described above for bacterial abundance (FACSCalibur, BD Biosciences). For viral counts, particles smaller than 0.5 µm (size fractions I and II) and smaller than 0.22 µm (category III) were distinguished. Only the smallest category was included into the analysis as it roughly corresponded to the diel cycle.

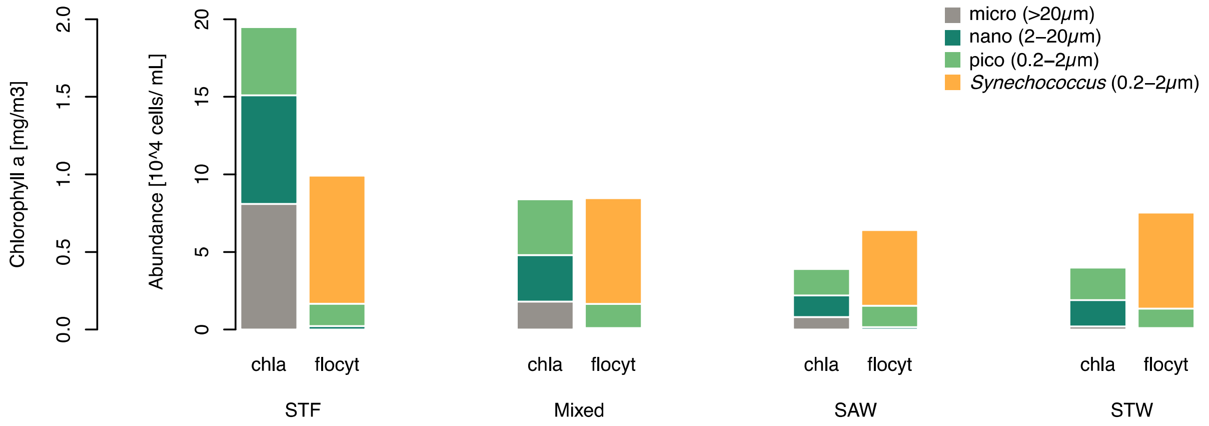

Figure S2. Phytoplankton community composition between the four regimes i.e. the Subtropical front (STF). mixed waters (Mix). Sub-Antarctic waters (SAW). and subtropical waters (STW) in chlorophyll a concentration (chla) and abundance data generated by flow cytometry (flocyt). Chlorophyll *a* fractions and abundance are distinguishing according to approximate size classes of phytoplankton cells.

Statistics

Due to the limited data available per time spot within each regime, the GAM model fit was based on an assumed normal distribution of the data. Three negative values of PCCHO were excluded before computing the GAM. It should be considered that while DOM release is likely followed by continuous consumption, its release could be steady (possibly the case in the afternoon with regards to DCCHO) or sudden (possibly the case at night, depending on the degree of synchronization of viral lysate pulses or grazing activity). In Figure 2a-d, we thus show the simplified and symmetric diel cycling of DOM as estimated curves. At least diel glucan anabolism and catabolism within the phytoplankton cell is symmetric [1] and corresponds to the approximated diel curves represented in Figure 2a, b.

Turnover calculations and its limitations

Turnover and the respective rates were calculated from the periodic diel shifts in DOM concentration, including both DCCHO and DAA. Therefore, the daily median minimum was subtracted from the daily median maximum. The estimated turnover is conservative as the minima and maxima of the natural diel cycles most certainly did not match our sampling schedule. Because the diel cycling of DCCHO and DAA was decoupled, the exact temporal distances between the maxima and minima were neglected and set to half a day (12 hours), which enabled us to calculate the pooled carbon and nitrogen turnover rates. Rates represent likewise release (positive rate) and degradation (negative rate).

We are aware of limitations in respect to how turnover rates were calculated here. In comparison to other studies but quantifying the particulate (intracellular) carbon pool, our time resolution was broader as it covered an eight-hour period instead of e.g. two to four hours [2, 3]. A shorter time period is certainly more relevant if diverse metabolic pathways of anabolism and catabolism within the

phytoplankton cell cycle are targeted. However, we intended to capture the well-known diel cycle of phytoplankton production [4].

The Sea2Cloud voyage was dedicated to resolve primary and secondary marine aerosol formation in relation to four different biogeochemical regimes, which we could unfortunately not monitor for the planned three weeks. After ten days, we had to return to Wellington harbor due to the Covid-19 pandemic lockdown. Yet, we were able to show a robust signal of DCCHO and DAA cycling despite of crossing diverse biogeochemical regions within short time. With regards to calculating DOM turnover, we compensated for the scarcity in data by relying on median instead of mean values and restricting our analysis to two regimes, in which at least three data points per time of day were available.

It should further be considered how TOC relates to DOC. The particulate organic carbon pool, comprising all living biomass, accounts for a minor fraction of TOC (~2%), the combined fraction of DOC (>1 kDa) accounts for already ~22% of TOC, while the remainder is truly dissolved [5]. It is thus reasonable to assume that TOC presents DOC fairly well and can be introduced as a proxy for DOC. In our calculation, carbon and nitrogen turnover is based on average TOC and TON concentrations, respectively. Especially in productive regimes, in which a higher faction of POC attributes to TOC, turnover is thus likely underestimated.

To avoid confusion, the terms *'turnover'* and its respective *'rates'* are defined exclusively quantitatively, while *'flux'* describes the movement of organic matter in and out of certain pools e.g. from the particulate to the dissolved and therewith deviates from its strict mathematical definition. In this study, *'cycling'* describes the overall process that organic matter pools are replenished and depleted within a periodic rhythm. *'Degradation'* is defined as the enzymatic breakdown of organic matter and/ or its transformation by microbes.

Degradation indices

While the degradation index (DI) is dependent on a subtle shift in the molecular composition of DAA [6, 7], the DAA-carbon yield (DAA-C yield) depends on the concentration of DAA [8]. To assign fractions of DAA concentration to the different stages of degradation, i.e. refractory (degraded within centuries), semi-labile (degraded within months to decades), and labile (degraded within hours to days), several assumptions are made [8]: 1) Because the microbial turnover of the refractory DAA pool is very slow (centuries), this fraction is basically the remainder after microbial degradation, its concentration is therefore stable, and well-mixed within the water column (surface to deep). By measuring the DAA concentration in very old (typically deep) waters, one can assume that the same, absolute refractory DAA concentration is also present in surface waters (~85.5 nM) [8]. 2) In contrast, microbial turnover of the labile DAA pool is very fast (hours to days), its concentration is highest at its place of production and release (the surface ocean), and therefore varies greatly in space and time. 3) The semi-labile DAA pool resides in-between those two opposing states. Regionally, it is rather well-mixed within the surface ocean until downwelling and seasonal mixing equilibrates its concentration with the deep water [9]. This relatively stable fraction

is assumed to be proportional to surface DOC concentrations [8]. Together with the refractory fraction, it represents 1.1-1.6% of DOC (DAA-C yield) [8]. Notably, all DAA fractions (refractory to labile) are characterized by differences in their molecular composition, i.e. the absolute DAA concentrations [nM] are not first-order correlated to their respective DAA-C yields [%]. By making these assumptions, and having surface DOC (here TOC) and DAA concentrations at hand, we could deduce the labile DAA fraction.

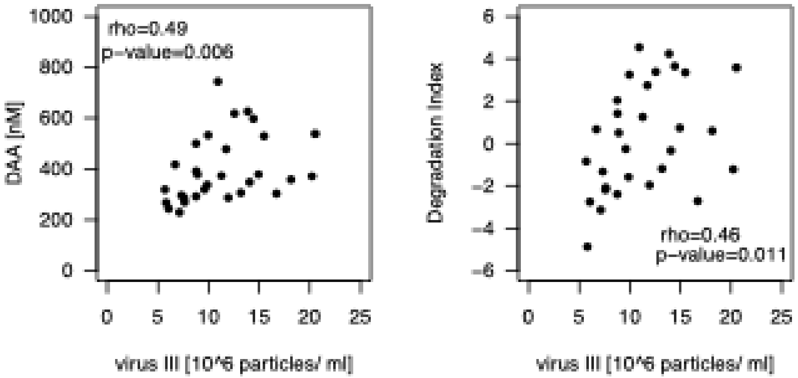

Figure S4. a) Dissolved amino acid (DAA) concentration in dependence of viral particle abundance (III). **b)** Degradation index in dependence of viral particle abundance. Non-parametric *Spearman* rank tests with the coefficient *rho* indicated a significant correlation.

DISCUSSION

Lability of cycled DAA

It has been established for decades that certain amino acids, such as GlX and Arg, are preferred over others in bacterial degradation experiments [10]. Three major bacterial groups dominate marine phytoplankton-associated communities of which one is specialized on the consumption of GlX, Arg, Leu, Iso, and Val [11]. During our campaign, an increase in the relative contribution of the same amino acids characterized the night-time DAA peak and defined the degradation index. In contrast, a relative increase of non-proteinogenous GABA has been associated with bacterial-driven organic matter decay [12] and relatively higher fractions of Ser, Gly, Ala characterize refractory organic matter profiles [6, 7, 12]. We could show that the degradation index in the morning was minimal and significantly differed from the degradation index at night. In incubation studies [12], the water column [6, 12], across seasons [13], and an oceanic front [14], reported changes in degradation indices were often less pronounced than the reoccurring diel shifts which we observed within eight hours and despite covering regimes of diverse biogeochemistry. As contrasting degradation indices and a decrease in labile substrate followed the night-time peak in DAA concentration, diel cycling of DAA and DCCHO can be attributed to rapid bacterial degradation.

Table S1. Number of samples depending on factors and parameters.

Variables	Sub-Antarctic water (SAW)	Subtropical water (STW)	Subtropical front (STF)	Mixed water (Mix)	afternoon (pm)	midnight (mi)	morning (am)
Chlorophyll *a*, TOC, AA and CCHO, DI (N=31)	n=10	n=3	n=12	n=6	n=10	n=10	n=11
TON (N=29)	n=9	n=3	n=11	n=6	n=10	n=8	n=11
Bacteria, virus particles (N=30)	n=9	n=3	n=12	n=6	n=10	n=10	n=10
Phytoplankton (N=29)	n=8	n=3	n=12	n=6	n=10	n=10	n=9

Table S2. Overview of the mean concentration and standard deviation of bulk parameters divided into factorial categories. Only significant *p*-values are displayed, values for which this was not applicable have been replaced by n/a. Abbreviations: total organic carbon (TOC), total organic nitrogen (TON), particulate carbohydrates (PCHO), particulate amino acids (PAA), dissolved combined carbohydrates (DCCHO), dissolved amino acids (DAA), afternoon (pm), midnight (mi), morning (am), subtropical front (STF), mixed regimes (Mix), sub-Antarctic waters (SAW), subtropical waters (STW).

Factor	**Time of the day**				**Regime**				
Category	pm	mi	am	art ANOVA	STF	Mix	SAW	STW	art ANOVA
Data format	*M ±SD*	*M ±SD*	*M ±SD*	*p-value <0.05*	*M ±SD*	*M ±SD*	*M ±SD*	*M ±SD*	*p-value <0.05*
TOC [µM]	81.1 ±10.9	76.4 ±15.0	76.7 ±10.4	na	88.4 ±8.4	74.4 ±2.4	68.8 ±12.3	74.7 ±1.5	0.0000
TON [µM]	7.11 ±2.11	6.76 ±1.30	6.73 ±1.27	na	8.24 ±1.24	6.28 ±0.47	5.66 ±1.42	6.65 ±0.39	0.0024
PCCHO [nM]	579 ±587	178 ±231	236 ±312	0.0344	632 ±552	199 ±202	96 ±69	124 ±91	0.0001
PAA [nM]	537 ±299	276 ±218	523 ±443	0.0092	737 ±352	324 ±232	233 ±104	176 ±25	0.0001
DCCHO [nM]	786 ±326	680 ±195	570 ±200	0.0100	903 ±261	517 ±64	528 ±79	607 ±221	0.0000
DAA [nM]	392 ±141	477 ±126	305 ±52	0.0006	437 ±157	436 ±109	348 ±105	290 ±26	0.0075
Degradation Index (DI)	-0.07 ±2.55	2.01 ±2.27	-1.57 ±1.22	0.0012	0.93 ±2.68	0.84 ±2.49	-0.27 ±2.05	-2.63 ±2.05	0.0375
Viral counts [10^6 particles mL^{-1}]	11.1 ±4.9	13.1 ±3.4	9.3 ±3.6	n/a	11.6 ±4.0	12.8 ±5.2	11.5 ±3.6	6.4 ±1.1	n/a
Bacteria [10^6 cells mL^{-1}]	2.67 ±0.92	2.67 ±1.23	2.70 ±0.40	n/a	3.30 ±0.84	2.75 ±0.90	2.05 ±0.379	1.71 ±0.41	0.0029
Synechococcus [10^3 cells mL^{-1}]	62 ±32	63 ±91	83 ±45	n/a	83 ±89	68 ±49	49 ±12	62 ±24	n/a
Picophytoplankton [10^3 cells mL^{-1}]	12.0 ±4.6	14.2 ±7.4	16.4 ±4.8	n/a	13.8 ±8.0	15.6 ±4.5	13.9 ±4.0	12.6 ±4.7	n/a
Nanophytoplankton [10^3 cells mL^{-1}]	1.43 ±0.80	1.60 ±0.79	1.77 ±84	n/a	2.23 ±0.64	0.88 ±0.36	1.43 ±0.60	0.87 ±0.06	0.0001
total Chlorphyll *a* [mg m^{-3}]	1.12 ±0.90	0.97 ±0.63	1.15 ±1.02	n/a	1.94 ±0.67	0.84 ±0.21	0.39 ±0.08	0.40 ±0.09	0.0001
Chlorophyll *a* 0.2-2µm [mg m^{-3}]	0.28 ±0.16	0.32 ±0.21	0.34 ±0.16	n/a	0.44 ±0.20	0.36 ±0.08	0.17 ±0.05	0.21 ±0.06	0.0000

Chlorophyll *a* 2-20µm [mg m^{-3}]	0.35 ±0.28	0.37 ±0.26	0.46 ±0.47	n/a	0.70 ±0.35	0.30 ±0.14	0.14 ±0.04	0.17 ±0.05	0.0001
Chlorophyll *a* >20µm [mg m^{-3}]	0.49 ±0.54	0.29 ±0.31	0.36 ±0.43	n/a	0.81 ±0.39	0.18 ±0.13	0.08 ±0.05	0.02 ±0.02	0.0000

Table S3. Overview of the concentration of the main dissolved amino acids (DAA) and main dissolved combined carbohydrates (DCCHO) contributing to diel carbon and nitrogen turnover separated by regimes.

Variable	**Dissolved amino acids (DAA)**				**Dissolved combined carbohydrates (DCCHO)**			
Time of day		pm	mi	am		pm	mi	am
Data format	[nM]	*M ±SD*	*M ±SD*	*M ±SD*	[nM]	*M ±SD*	*M ±SD*	*M ±SD*
Subtropical front (STF)	glutamic acid (GlX)	80 ±50	87 ±14	31 ±9	glucose (Glc)	649 ±195	461 ±73	323 ±250
(n=12)	glycine (Gly)	111 ±28	122 ±13	73 ±10	mannose/xylose (ManXyl)	163 ±11	172 ±16	135 ±17
	aspartic acid (AsX)	82 ±21	90 ±9	47 ±10	fucose (Fuc)	63 ±8	60 ±7	47 ±15
	alanine (Ala)	57 ±21	61 ±7	33 ±6	glucosamine (GlcN)	51 ±4	44 ±6	44 ±12
	arginine (Arg)	26 ±13	30 ±6	11 ±3	galactose (Gal)	71 ±4	64 ±7	60 ±5
	leucine (Leu)	19 ±8	23 ±3	8 ±2				
Mixed water (Mix)	glutamic acid (GlX)	55 ±18	98 ±22	42 ±15	glucose (Glc)	146 ±25	219 ±53	190 ±17
(n=6)	glycine (Gly)	99 ±8	119 ±9	90 ±13	mannose/xylose (ManXyl)	96 ±6	124 ±24	122 ±3
	aspartic acid (AsX)	62 ±0	91 ±5	62 ±11	fucose (Fuc)	39 ±3	38 ±3	39 ±5
	alanine (Ala)	45 ±0	65 ±3	45 ±9	glucosamine (GlcN)	47 ±2	40 ±4	45 ±11
	arginine (Arg)	15 ±1	17 ±1	10 ±1	galactose (Gal)	55 ±5	54 ±6	56 ±7
	leucine (Leu)	11 ±2	25 ±2	13 ±7				
Sub-Antarctic w. (SAW)	glutamic acid (GlX)	36 ±5	57 ±27	33 ±1	glucose (Glc)	167 ±29	185 ±84	91 ±20
(n=10)	glycine (Gly)	80 ±9	100 ±27	76 ±5	mannose/xylose (ManXyl)	128 ±15	138 ±10	127 ±16
	aspartic acid (AsX)	52 ±10	68 ±20	51 ±3	fucose (Fuc)	39 ±3	39 ±6	33 ±1
	alanine (Ala)	36 ±5	48 ±17	36 ±1	glucosamine (GlcN)	50 ±3	47 ±6	46 ±2
	arginine (Arg)	14 ±1	23 ±10	13 ±2	galactose (Gal)	70 ±8	67 ±7	65 ±3
	leucine (Leu)	11 ±4	16 ±9	10 ±2				
Subtropical w. (STW)	glutamic acid (GlX)	24	34	37	glucose (Glc)	450	128	160
(n=3)	glycine (Gly)	72	73	82	mannose/xylose (ManXyl)	125	124	112
	aspartic acid (AsX)	43	47	55	fucose (Fuc)	51	41	36
	alanine (Ala)	40	38	39	glucosamine (GlcN)	48	39	41
	arginine (Arg)	4	6	12	galactose (Gal)	76	56	55
	leucine (Leu)	9	9	12				

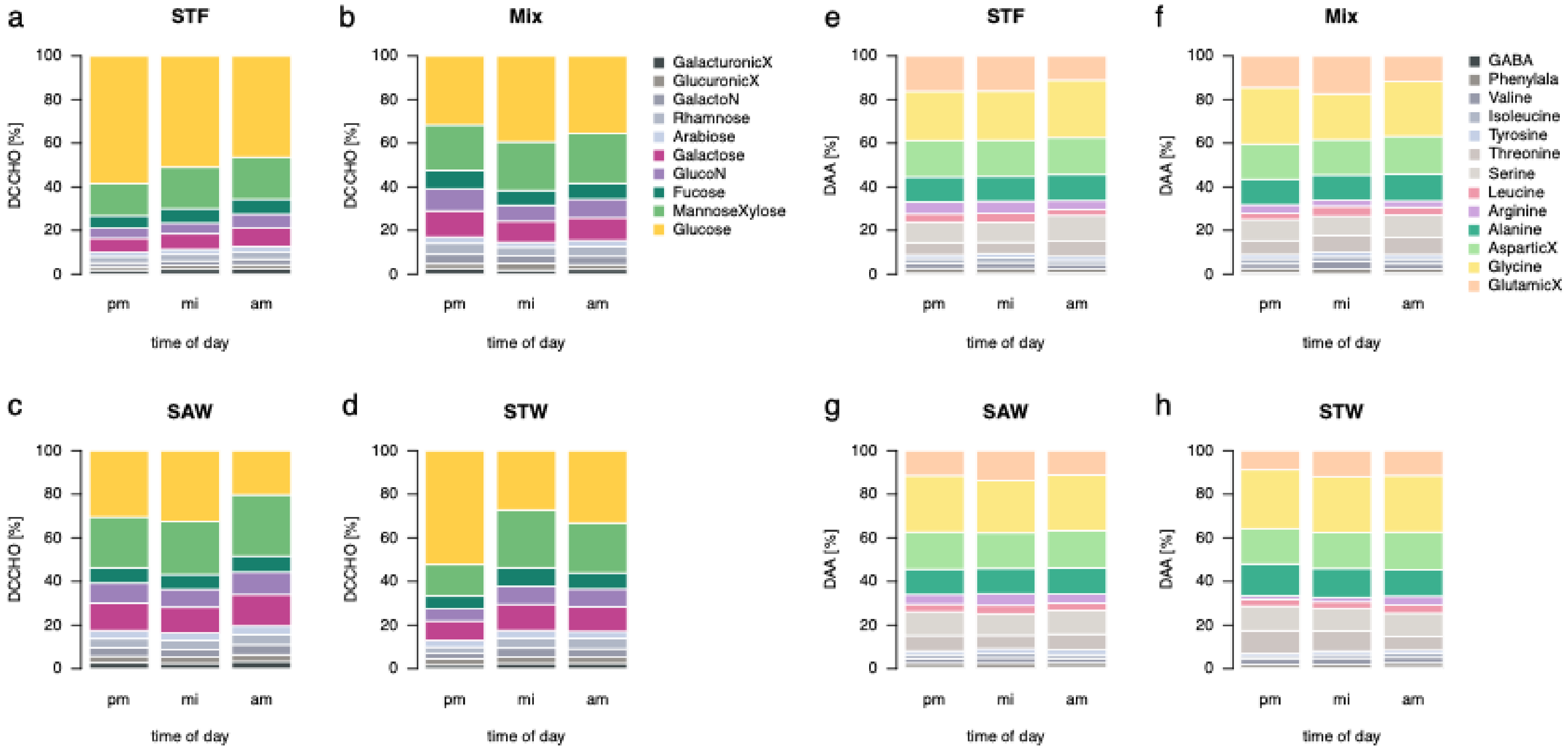

Figure S3. Molecular composition in percent of **a-d)** dissolved combined carbohydrates (DCCHO) and **e-h)** dissolved amino acids (DAA) over the course of the day. Colors represent the molecular components which contributed most to diel turnover, while dark to light gray represent the remaining components. In the upper panels, the two regimes with replete nutrient concentrations are depicted and comprise the subtropical front (STF) and the mixed waters (Mix). Lower panels show the deplete regimes, including the sub-Antarctic (SAW) and subtropical waters (STW). Abbreviations: Afternoon (pm); Midnight (mi); Morning (am).

Table S4. Overview representing numbers used to calculate diel turnover (X) and turnover rates (d(n)/ d(t)) from maximal and minimal concentrations of dissolved free and hydrolysable amino acids (DAA) and dissolved combined carbohydrates (DCCHO) in two regimes of the Southwestern Pacific Ocean i.e. the subtropical front (STF) and sub-Antarctic waters (SAW). DCCHO turnover rates are also represented in glucose (Glc) equivalents. Comparative literature values are listed below.

	Time	Regime	Concentrations		Turnover rates			Literature	Turnover	
			[µM C]	[µM N]	[nM C h^{-1}]	[nM N h^{-1}]	[nM Glc h^{-1}]		C %	N %
min DAA	am	SAW	1.751	0.405						
max DAA	mi	SAW	2.256	0.548						
delta DAA	8 hours	SAW	0.505	0.143	63.2	18.0		a, b, c		
min DAA	am	STF	1.606	0.370						
max DAA	mi	STF	3.204	0.808						
delta DAA	8 hours	STF	1.598	0.438	199.7	54.7		a, b, c		
min DCCHO	am	SAW	2.765	0.064						
max DCCHO	pm	SAW	3.323	0.075						
delta DCCHO	16 hours	SAW	0.558	0.011	34.9	0.7	5.8	d, e		
min DCCHO	am	STF	4.041	0.053						
max DCCHO	pm	STF	6.831	0.066						
delta DCCHO	16 hours	STF	2.790	0.013	174.3	0.8	29.1	d, e		
delta sum (DCCHO, DAA)	12 hours	SAW	1.063	0.154	88.6	12.9				
delta sum (DCCHO, DAA)	12 hours	STF	4.388	0.451	365.6	37.5				
TOC/TON (M ±SD)		SAW	68.8 ±12.3	8.2 ±1.2					1.6	2.7
TOC/TON (M ±SD)		STF	88.4 ±8.4	5.7 ±1.4					5.0	5.5

a Uptake rates of dissolved free amino acids: 3.8-35.3 nM N h^{-1}, Fuhrman, (1987)
b Uptake rates of dissolved combined amino acids: 6.6-28.3 nM N h^{-1}, Rosenstock & Simon (1993)
c Further uptake rates of DAA reviewed by Berman & Bronk (2003)
d Laminarin degradation rates in Glc monomers: 1.6-34 nM Glc h^{-1}, as reviewed by Becker et al., (2020)
e Laminarin hydrolysis rates in Glc monomers: max. 20-22 nM Glc h^{-1}, Reintjes et al., (2019)

REFERENCES

(1) Van Oijen, T.; Van Leeuwe, M. A.; Granum, E.; Weissing, F. J.; Bellerby, R. G. J.; Gieskes, W. W. C.; De Baar, H. J. W. Light rather than iron controls photosynthate production and allocation in Southern Ocean phytoplankton populations during austral autumn. *Journal of Plankton Research* **2004**, *26* (8), 885-900. DOI: 10.1093/plankt/fbh088.
(2) Boysen, A. K.; Carlson, L. T.; Durham, B. P.; Groussman, R. D.; Aylward, F. O.; Ribalet, F.; Heal, K. R.; White, A. E.; DeLong, E. F.; Armbrust, E. V.; Ingalls, A. E. Particulate Metabolites and Transcripts Reflect Diel Oscillations of Microbial Activity in the Surface Ocean. *mSystems* **2023**, *8* (3). DOI: 10.1128/msystems.01083-22.
(3) Muratore, D.; Boysen, A. K.; Harke, M. J.; Becker, K. W.; Casey, J. R.; Coesel, S. N.; Mende, D. R.; Wilson, S. T.; Aylward, F. O.; Eppley, J. M.; Vislova, A.; Peng, S.; Rodriguez-Gonzalez, R. A.; Beckett, S. J.; Virginia Armbrust, E.; DeLong, E. F.; Karl, D. M.; White, A. E.; Zehr, J. P.; Van Mooy, B. A. S.; Dyhrman, S. T.; Ingalls, A. E.; Weitz, J. S. Complex marine microbial communities partition metabolism of scarce resources over the diel cycle. *Nature Ecology and Evolution* **2022**, *6* (2), 218-229. DOI: 10.1038/s41559-021-01606-w.
(4) Halsey, K. H.; Jones, B. M. Phytoplankton Strategies for Photosynthetic Energy Allocation. *Annual Review of Marine Science* **2015**, *7* (1), 265-297. DOI: 10.1146/annurev-marine-010814-015813.
(5) Benner, R.; Amon, R. M. W. The Size-Reactivity Continuum of Major Bioelements in the Ocean. *Annual Review of Marine Science* **2015**, *7* (1), 185-205. DOI: 10.1146/annurev-marine-010213-135126.
(6) Kaiser, K.; Benner, R. Biochemical composition and size distribution of organic matter at the Pacific and Atlantic time-series stations. *Marine Chemistry* **2009**, *113* (1-2), 63-77. DOI: 10.1016/j.marchem.2008.12.004.
(7) Dauwe, B.; Middelburg, J. J.; Herman, P. M. J.; Heip, C. H. R. Linking diagenetic alteration of amino acids and bulk organic matter reactivity. *Limnology and Oceanography* **1999**, *44* (7), 1809-1814. DOI: 10.4319/lo.1999.44.7.1809.
(8) Davis, J.; Benner, R. Quantitative estimates of labile and semi-labile dissolved organic carbon in the western Arctic Ocean: A molecular approach. *Limnology and Oceanography* **2007**, *52* (6), 2434-2444. DOI: 10.4319/lo.2007.52.6.2434.
(9) Hansell, D. A. Recalcitrant dissolved organic carbon fractions. *Annual Review of Marine Science* **2013**, *5*, 421-445. DOI: 10.1146/annurev-marine-120710-100757.
(10) Amon, R. M. W.; Fitznar, H. P.; Benner, R. Linkages among the bioreactivity, chemical composition, and diagenetic state of marine dissolved organic matter. *Limnology and Oceanography* **2001**, *46* (2), 287-297. DOI: 10.4319/lo.2001.46.2.0287.
(11) Ferrer-González, F. X.; Widner, B.; Holderman, N. R.; Glushka, J.; Edison, A. S.; Kujawinski, E. B.; Moran, M. A. Resource partitioning of phytoplankton metabolites that support bacterial heterotrophy. *ISME Journal* **2021**, *15* (3), 762-773. DOI: 10.1038/s41396-020-00811-y.
(12) Davis, J.; Kaiser, K.; Benner, R. Amino acid and amino sugar yields and compositions as indicators of dissolved organic matter diagenesis. *Organic Geochemistry* **2009**, *40* (3), 343-352. DOI: 10.1016/j.orggeochem.2008.12.003.
(13) Barthelmeß, T.; Engel, A. How biogenic polymers control surfactant dynamics in the surface microlayer: insights from a coastal Baltic Sea study. *Biogeosciences* **2022**, *19* (20), 4965-4992. DOI: 10.5194/bg-19-4965-2022.

(14) Barthelmeß, T.; Schütte, F.; Engel, A. Variability of the Sea Surface Microlayer Across a Filament's Edge and Potential Influences on Gas Exchange. *Frontiers in Marine Science* **2021**, *8* (October), 1-21. DOI: 10.3389/fmars.2021.718384.
